# Zero x-rays radiofrequency catheter ablation for ventricular premature contraction originating from the left coronary cusp during pregnancy: a case report

**DOI:** 10.3389/fcvm.2023.1183787

**Published:** 2023-09-08

**Authors:** Changjin Li, Haoyu Gu, Chao Liu, Ke Li, Xianbu Gao, Manli Yu, Zhifu Guo

**Affiliations:** Department of Cardiovascular Medicine, Changhai Hospital, Naval Medical University, Shanghai, China

**Keywords:** zero x-rays, radiofrequency, catheter ablation, ventricular premature, left coronary cusp

## Abstract

Pregnancy predisposes to arrhythmias in females due to physiological changes in the cardiovascular system, enhanced activity of the sympathetic nervous system (SNS), and changes in the endocrine system, regardless of whether there exist cardiovascular diseases before the pregnancy. Tachyarrhythmias may present for the first time or worsen persistently during pregnancy, potentially leading to maternal heart failure and sudden death, as well as some adverse fetal outcomes such as growth restriction, distress, premature birth, and stillbirth. Radiofrequency ablation (RFA) is one of the most important therapeutic methods for tachyarrhythmias. According to the 2019 European Society of Cardiology (ESC) guidelines, RFA in pregnant women should preferably be performed without x-rays. Since the 2000s, 3D mapping technique has rapidly developed, laying the foundation for cardiac electrophysiology examination free from x-rays. Ventricular arrhythmia originating from the left coronary cusp (LCC) is not common in clinic. RFA is challenging in the treatment of this type of disease due to the anatomical feature that the opening of the left main coronary artery is localized in the LCC.

## Introduction

The Ensite NavX three-dimensional (3D) mapping system can visually construct the 3D cardiac model by the catheter was moved in region of heart, with the left main opening and ablation position marked, conducive to increasing the success rate and safety of surgery. Here, we report a case of a pregnant woman, who underwent zero x-rays RFA for ventricular premature contraction (VPC) originating from the LCC under the guidance of the Navix 3D mapping system, and obtained good results.

## Case presentation

**Figure 1:**
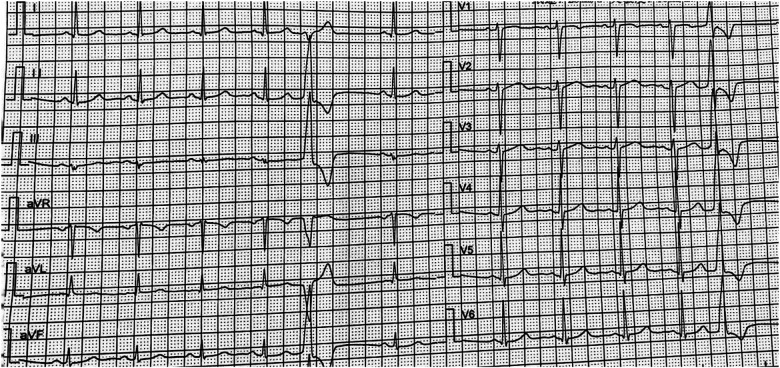
ECG showed the VPC: the QRS complex was RS type in lead V1, migrated to lead V2. The limb lead I was rS type of QRS complex, and R type was showed in II, III, and avF limb leads, which was considered that the VPC may originate from the left coronary sinus.

A 25-year-old woman was admitted for a 2-year history of recurrent palpitation and 6-month exacerbation. She did not have children and was pregnant (12 weeks of gestation) on admission. Symptoms occurred both before and during the pregnancy. 12-lead electrocardiogram (ECG) during pregnancy revealed sinus rhythm with frequent VPC bigeminy (Figure 1). The patient had no significant past medical history. On admission physical examination, she had a clear mind and normal spirit. The blood pressure (BP) was 128/75 mmHg, and the heart rate (HR) was 80 beats/min. Irregular heartbeat was found as VPC bigeminy without valvular murmurs. Laboratory examination showed the B-type natriuretic peptide (BNP) of 103.93 pg/ml (<100 pg/ml), myoglobin (Myo) of 10.40 ng/ml (14.3–65.8 ng/ml), plasma D-dimer of 1.24 ug/ml (<0.55 mg/L), and normal other parameters.

Transthoracic echocardiography (TEE) revealed 63% of left ventricular ejection fraction (LVEF), left atrium 38 mm (<38 mm), interventricular septal thickness (IVST) 9 mm (8–15 mm), no structural abnormality of heart was found. The 24 h Holter ECG recorded 95,275 total heartbeats, HR 65 bpm on average (47–136 beats/min), and 20,000 total VPCs. No antiarrhythmic drug was used before the procedure.

The patient refused alternative treatment such as conservative treatment. After multidisciplinary discussion, zero x-rays RFA was recommended for the treatment. Written informed consent was obtained. Before the procedure, the patient was placed in supine position, connecting to an electrophysiological recording system, the stimulator of the ECG, an RFA device, an electrical defibrillator, the positioning electrode of the Navx 3D mapping system, and a dorsal electrode. ECG revealed VPC originating from the LCC. During the procedure, the site of puncture was firstly routinely disinfected, followed by spreading of sterile surgical towels. After local anesthesia using 1% Novocaine, the left femoral vein was punctured, and a 7F venous sheath was inserted to send a 10-polar electrode to the coronary cusp and a 4-polar electrode to the right ventricular apex. The test result showed RV S1S1 600–400 ms, RVS1S1 600 ms, YA conduction block in a Wenckebach pattern, His A-wave in the lead; RA S1S1 500–250 ms, RA S1S1 300 ms, AV conduction block in a Wenckebach pattern, His V-wave in the lead; RAS1S2 500/300 ms, inverse step size 10 s, AVNERP < 500/250 ms.

**Figure 2:**
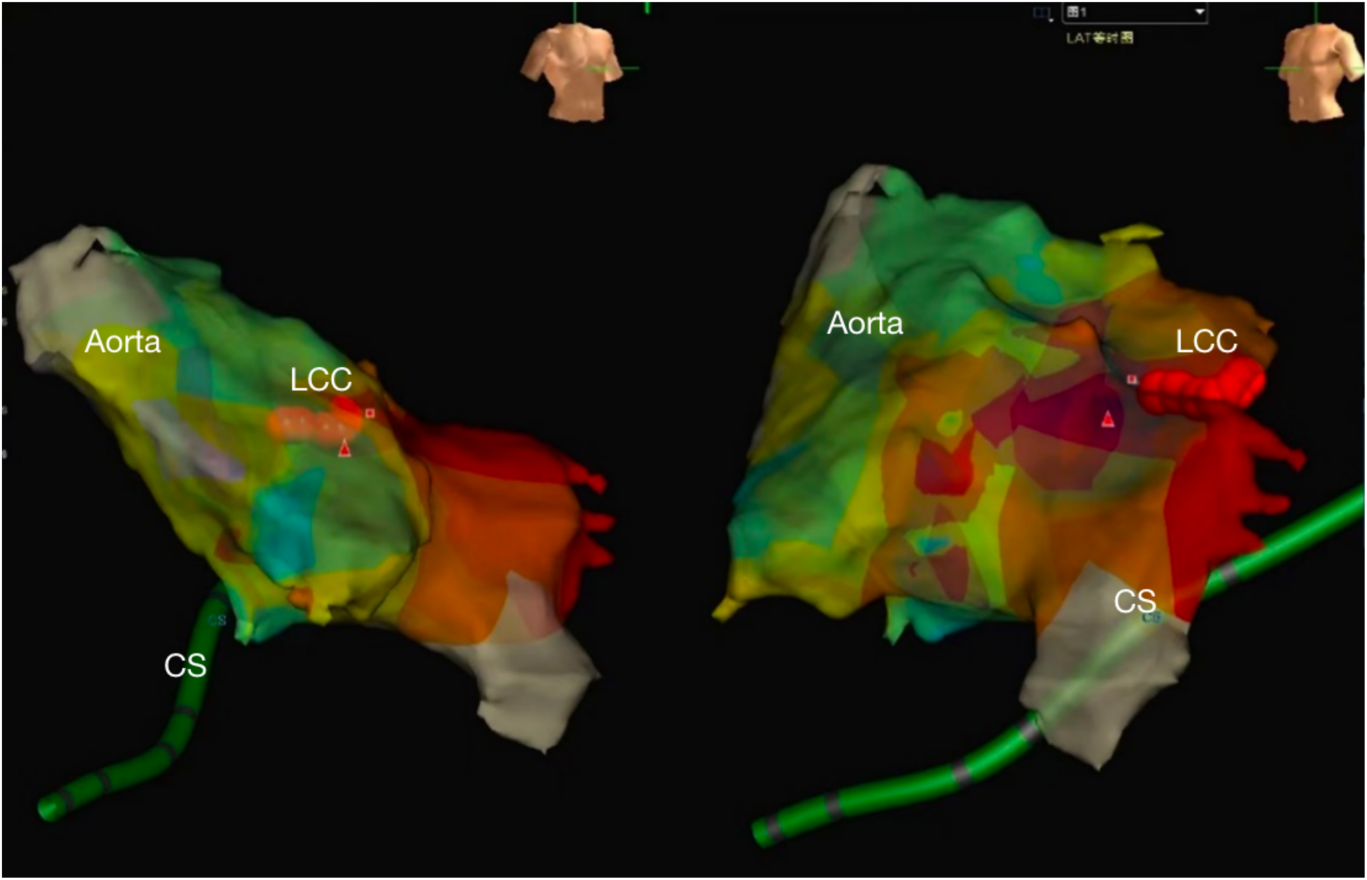
The NavX three-dimensional (3D) mapping system showed the local aorta and left ventricle, the successive red points were ablation target located the left coronary sinus. (CS, coronary sinus, LCC, left coronary cusp).

**Figure 3:**
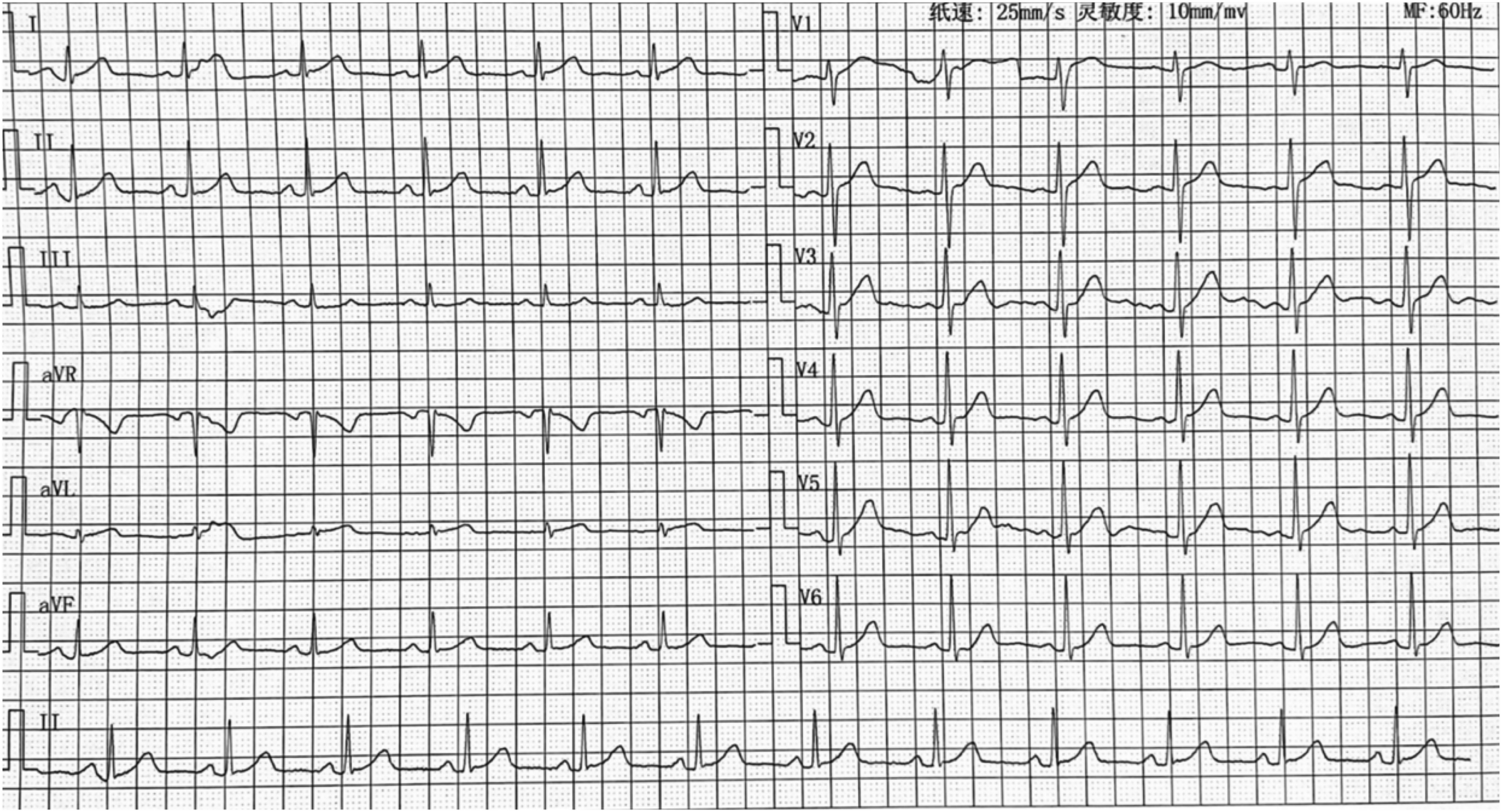
ECG showed normal sinus rhythm after radiofrequency ablation.

The right femoral artery was then punctured under local anesthesia with 1% Novocaine, and an 8F arterial sheath was inserted followed by injection of 32 mg heparin. A cold saline solution-irrigated ablation catheter (IBI) was inserted to the LCC through the arterial sheath, targeting the site 0.5 cm beneath the anterior opening of the left coronary artery (Figure 2). Activation mapping showed that local potential preceded the surface QRS wave by approximately 10 ms (RF = 43°C, radio frequency power = 25 W), and the VPC disappeared after firing. There was no VPC in the following 30 min. After completion of the procedure, the venous sheath and femoral arterial sheath were removed successively. The femoral artery was closed using the Proclose suture instrument, followed by pressure dressing. A 12-lead ECG was performed immediately and on day 2 after the procedure, suggesting sinus rhythm without VPC (Figure 3). No arrhythmia was found during ECG monitoring. The patient was discharged on antiplatelet therapy with aspirin 100 mg/d for 1 month. At 2 weeks postoperatively, the patient had no palpitation, chest tightness, or other discomfort.

## Discussion

In normal pregnant women, there are a series of changes in the cardiovascular system and uterus (increased uterine volume and gradually elevated uterine fundus), diaphragmatic ascent that leads to upward and forward displacement of the heart, and mild torsion of great vessels (1). In addition, the blood volume and cardiac output during pregnancy increase, and there is a compensatory increase in HR that leads to sinus tachycardia and various premature contractions in some of the women. Moreover, the adverse events in early pregnancy, such as nausea, vomiting, and fewer meals, lead to water and electrolyte imbalance and a potential increase in the incidence of arrhythmia (2, 3). Pregnancy with complicated arrhythmia commonly presents with symptoms, such as chest tightness and palpitation, in clinical practice, while the severity of these symptoms is dependent on the effect of arrhythmias on hemodynamics. If there is a reduction in ventricular filling due to arrhythmia, the cardiac output will be decreased, resulting in symptoms such as chest tightness and palpitation. While upon a severe reduction in cardiac function, the inadequate left ventricular filling but increased resistance of pulmonary circulation can result in left heart failure. In addition, arrhythmia can significantly increase the risk of maternal death, and its high rate of recurrence causes a higher risk for maternal-fetal adverse events. Tachyarrhythmias may present for the first time during pregnancy or worsen persistently, potentially leading to maternal heart failure and sudden death, and some adverse fetal outcomes such as growth restriction, distress, premature birth, and stillbirth (1). Therefore, there is a need for close monitoring during pregnancy to provide timely interventions to reduce severe maternal and perinatal complications (1). Presently, drug therapy remains the first-line treatment for arrhythmia during pregnancy in the acute phase and for a long-term period (4). However, it is limited due to the potential effect of antiarrhythmic drugs on fetus. Some physical approaches, such as transesophageal atrial pacing (TAP) and electrical cardioversion, are reproducible and easy to operate, but there are some drawbacks including low efficiency and easy recurrence within a short time. Radiofrequency catheter ablation (RFCA) is recommended by guidelines in the context of poor drug efficacy or presence of VPC refractory to medications. Indications for cardiac RFCA include supraventricular tachycardia, atrial tachycardia, partial atrial fibrillation, VPC, idiopathic ventricular tachycardia with normal cardiac structure and function, and bundle branch reentrant tachycardia (BBRT). Contraindications for cardiac RFCA are atrial thrombus and abnormal coagulation (5). The conventional x-rays RFA has a risk of inducing radiographic damage, and it may cause fetal cancer in pregnant women even with low-dose x-rays (10 mGy). The patient reported here had severe symptoms. Drug therapy was not recommended, while zero x-rays RFCA under the guidance of a 3D mapping system was considered. Currently, zero x-rays RFCA for treatment of VPC originating from the LCC has not yet been reported.

VPC is among the most common arrhythmias in clinical practice that can originate from various parts of the ventricles, among which the left/right ventricular outflow tracts (LVOT/RVOT) are the most common sites (6). Frequent VPC can lead to arrhythmogenic cardiomyopathy (AC). Therefore, a radical cure of VPC is of great clinical significance. The VPC originating from LVOT/RVOT has typical ECG changes, including tall abnormal R-waves in the QRS complex in the inferior wall leads, and common complete left bundle branch block (LBBB) in the precordial leads [partial right bundle branch block (RBBB)]. In recent years, RFCA has been extensively applied in the clinical treatment of LVOT/RVOT-VPC, and the success rate is up to 90% (7). Comparatively, RFCA is more effective for the RVOT-VPC with a lower risk (8), which might be due to the more complex anatomical structure of the LVOT that increases the difficulty in localizing VPC. The LVOT is located in the middle heart and comprises four parts: LCC, right coronary cusp (RCC), non-coronary cusp (NCC), and subvalvular myocardial tissues. The LVOT is adjacent to the RVOT and located approximately to the right rear of the RVOT. The QRS morphology in precordial leads, especially in leads V1 and V2, is key to distinguishing between the VPC originating from the LVOT and RVOT. QRS waves with the following characteristics are highly suggestive of LVOT-VPC: (1) RBBB in lead V1; (2) QRS wave in precordial lead prior to lead V2; (3) R-wave amplitude in lead V1 > lead V2; (4) a higher R-wave amplitude (>30% of the total QRS wave amplitude) and a wider time-course (>50% of the total QRS wave time course) in lead V1 (9). The aortic valve within the LVOT involves LCC, RCC and NCC. LCC is the highest (top left) adjacent to the left atrium and pulmonary arterial root; RCC is located to the crest of the muscular part of the interventricular septum (front right), connecting the right atrium and ventricle and adjacent to the RVOT via the conidial septation; while NCC is located underneath the posterior aortic valve, adjacent to the right and left atria.

The VPC originating from the LCC also has specific ECG findings: right-skewed axis; high-amplitude R-waves in leads Ⅱ, Ⅲ and aVF; leads I and aVL dominated by S-waves; R/S > 1 in lead V3; high-amplitude R-waves and absence of S-wave in leads V5 and V6 (10); largely varied QRS morphology in lead V1 (e.g., RS, Rs and rS). However, the rS pattern in lead V1 predisposes to misdiagnosis with the VPC from the RCC. It is helpful to identify the VPC originating from the RCC if the following ECG findings are revealed: the time course of R-wave is wider than 1/2 time course of QRS wave, or the ratio of R/S-wave amplitude is greater than 30% in lead V1 (11); lead V3 is dominated by R-waves.

RFA for the VPC originating from the LCC is usually performed under the guidance of an activation mapping system to find the earliest activation (12). Since the opening of the left main coronary artery is localized in the LCC, care should be taken to avoid damage to the left main trunk during ablation. In addition, the target for ablation is generally devised to the site over 10 mm away from the left main trunk to ensure safety, which can be determined by routine coronary angiography before ablation. The Navix mapping system can intuitively mark the target for ablation and the opening of the left main trunk on a 3D structural map. Moreover, it provides real-time location of the ablation positions, increasing the procedural safety and effectiveness.

To conclude, zero x-rays RFCA was a safe and effective strategy for severe cardiac arrhythmias in pregnant women. A reasonable prediction of VPC origin, solid intracardiac electrophysiological knowledge and skillful techniques of the operators, and the efficient cooperation with mapping technicians are critical to the success of zero x-rays RFCA.
